# Supplementary material for: Improving Oral Health with Fluoride-Free Calcium-Phosphate-Based Biomimetic Toothpastes: An Update of the Clinical Evidence
Source: Biomimetics (Basel). 2023 Jul 27;8(4):331. doi: 10.3390/biomimetics8040331 (PMC10452078; doi:10.3390/biomimetics8040331)
Supplement: Supplementary file 1 [file biomimetics-08-00331-s001.zip › biomimetics-2492826-supplementary.pdf]

Table S1. Search results using designated search terms

Search terms	Database	"hydroxyapatite", or "nano-hydroxyapatite" or "hydroxylapatite"	"ACP" or "Amorphous Calcium Phosphate" or "CPP-ACP"	"Calcium sodium phosphosilicate" or "CSPS" or "Bioglass" or "Novamin"	"beta-tricalcium phosphate" or "β-TCP" or "tricalcium phosphate" or "TCP"	Totals
+ Vehicle: "toothpaste" or "dentifrice"	PubMed<br>Scopus<br>Web of science<br>Google Scholar	625<br>1155<br>213<br>21,921	677<br>478<br>120<br>11,720	312<br>296<br>103<br>6426	134<br>170<br>33<br>10,088	1748<br>2099<br>469<br>not included
+ Experimental: "In vivo" or "In situ" or "clinical trial" or "Clinical study" or "Randomized clinical trial"	PubMed<br>Scopus<br>Web of science<br>Google Scholar	71<br>448<br>64<br>6440	122<br>294<br>60<br>257	31<br>165<br>64<br>2279	19<br>83<br>17<br>4500	243<br>990<br>205<br>not included
+ Outcome (Remineralization) "caries" or "white spot lesion" or "WSL", "remineralization", "erosion"	PubMed<br>Scopus<br>Web of science<br>Google Scholar	315<br>46<br>40<br>13,660	108<br>66<br>57<br>5200	7<br>9<br>26<br>2860	12<br>15<br>15<br>2341	442<br>136<br>138<br>not included
+ Outcome (Dentin Hypersensitivity): "sensitivity" or "hypersensitivity"	PubMed<br>Scopus<br>Web of science<br>Google Scholar	26<br>239<br>22<br>5470	41<br>62<br>3<br>2038	91<br>146<br>49<br>2656	3<br>22<br>0<br>1778	161<br>469<br>74<br>not included
+ Outcome (Whitens): "whiten" or "whitens" or "whitening"	PubMed<br>Scopus<br>Web of science<br>Google Scholar	64<br>87<br>4<br>4505	12<br>23<br>0<br>5328	8<br>12<br>3<br>2514	3<br>4<br>1<br>1204	87<br>126<br>8<br>not included
+ Outcome (Gingival health): "gingival" or "gingivitis" or "periodontal" or "periodontitis"	PubMed<br>Scopus<br>Web of science<br>Google Scholar	44<br>213<br>9<br>35,400	34<br>52<br>2<br>21,000	61<br>84<br>10<br>8332	10<br>20<br>1<br>8643	149<br>369<br>22<br>not included
